# Virtual Support in Dementia: A Possible Viable Strategy for Caregivers

**DOI:** 10.3389/fneur.2021.662253

**Published:** 2021-08-13

**Authors:** Ceres Ferretti, Ricardo Nitrini, Sonia M. D. Brucki

**Affiliations:** Cognitive and Behavioral Neurology Group, Faculty of Medicine, Hospital das Clínicas, University of São Paulo, São Paulo, Brazil

**Keywords:** dementia, support virtual, education health, nursing, care, caregiver

## Abstract

**Background:** In the last 10 months, due to the Covid-19 pandemic, several studies have shown that health education and virtual support strategies for caregivers of patients with dementia, in the management of home care, can be viable. Low and middle income countries, in particular, have sought to use these means to reduce the daily burden of caregivers, through virtual meetings of education and support.

**Objectives:** To present the feasibility of a pilot study on the use of a support action contemplated by the Caad Project–indirect costs of dementia–from HC-FMUSP.

**Methods:** Observational study in which 93 caregivers were invited to participate in virtual meetings on a frequency of three times/week, lasting 1 h each.

**Results:** Of the 93 invited family members, and after 3 months, 42 answered eight questions about the effectiveness of the action. High percentages of positive responses regarding program satisfaction ranged from 86 to 100%.

**Conclusion:** This study showed results of a very simple intervention that suggests that it is possible to offer caregivers of patients with dementia a program that can be used in primary care, in order to understand the difficulty of caregivers in their daily care of patients with dementia, with daily management guidelines on a case-by-case basis, in addition to promoting the implementation of an education strategy about the importance of knowing, and recognizing anatomophysiological changes in the aging process and its implications for the rupture of the imaginary line that involves senescence and senility. This allows the caregiver to feel able to protect his patient and himself by preventing the emergence of common diseases in this age group. Further studies are needed to explore this type of non-pharmacological support.

## Introduction

Never, in history, has there been a greater need for adaptation and social change than that seen today. The world is perplexed, facing the biggest health crisis of all time amid the pandemic caused by COVID-19. Low-, middle-, and high-income countries (LMIC) find themselves totally unprepared, both economically and socially, to prevent more people every day from becoming infected and requiring hospital support and beds in high complexity services, which are simply not available to all those in need. Doctors and nurses on the frontline are suffering the mental impact of the pandemic, while also themselves vulnerable to contamination and exposed to a life-threatening situation (1, 2). A recent study presented alarming data related to the number of people affected by the new coronavirus pandemic, which has surpassed 10.5 million people infected worldwide (3). Today, we remain perplexed by the worsening of this scenario as epidemiological reports present even more alarming figures showing how the second wave of COVID-19 is already plaguing Europe and the USA, each with 52,593,188 and 32,210,817 confirmed cases, respectively. Thus, the mark of more than 155,665,214 million cases globally has been reached, ~2% of the world population, with 3,250,648 related deaths (4).

For Latin American countries, the pandemic involving the new coronavirus has had an even more negative impact, due to the limited resources available regarding the health system and society as a whole. These factors meant the global health crisis has taken on a much greater dimension, as it affects thousands of people who do not have equal access to health services (5). At this time, Brazil is concerned over the soaring number of cases signaling the beginning of the second wave. Current data confirm more than 14,930,183 cases of infection by the disease and over 414,399 related deaths (4, 6). Researchers and health professionals are striving for science to prevail and for protection and prevention measures to be adopted by all, a challenge that has proven far from easy. The country has many cultural differences and socio-economic inequalities that facilitate the spread of the virus since not all regions and populations adhere to the health and hygiene measures recommended by the WHO (5, 7). In some cases, this is because the physical conditions and housing preclude adherence, while for others, there is resistance to adopting the necessary measures, such as the use of the main protective equipment, the face mask. In any event, most societies are committed to finding ways to prevent further spread of the disease. For 10 months, we have been following the alarming devastation caused by a virus about which very little is known, yet forces thousands of people to remain isolated in their homes (8). Never before have so many nations come together in an attempt to reduce the number of victims and attenuate the social and financial impact that have led to the crash in the world economy (9). Everyone is unanimous in stating that only the vaccine can have a positive impact and even though doubts remain about possible immunity, scientists agree this is the only way to control the spread and restore community life. This “new normal” seems to be the best alternative until we are sure, as occurred for the H1N1 flu epidemic, that we will indeed achieve immunity, albeit temporary or permanent (9). Some chronic non-communicable diseases (CNCDs), such as dementia with different etiologies, require coping strategies be developed in LMICs, given the clinical course of patients and high morbidity associated with these processes (3, 4). Virtual support programs seem promising and simple, low-cost non-pharmacological interventions have shown positive results (10). This pilot study sought to determine the utility of a virtual non-pharmacological education and support program for caregivers of dementia patients.

**Figure 1:**
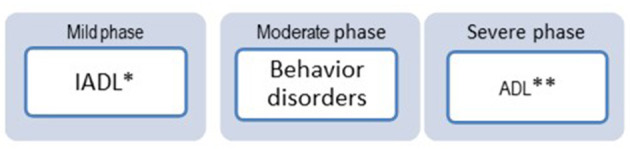
Dependencies associated with the evolution stages of Ferretti (2020). ^*^IADL, Instrumental activities daily living; ^**^, ADL, activities daily living.

Evidence in the literature clearly shows that multi- and interdisciplinary non-pharmacological approaches are always the first line of care for patients with functional and behavioral complaints (11–13). Alternatively, combined therapy (14) can also be useful, and professionals involved in caring for people with dementia and their families need to be well-prepared with regard to their knowledge on the peculiarities encountered in the progression of different types dementia, with a view to proposing and developing an effective care plan on a case-by-case basis (12) and following guidelines addressing the disease in a focused and individualized way, meeting the inherent needs at each stage (Figure 1).

One of the basic and essential responsibilities in nurse training is the work that involves health promotion and disease prevention, i.e., health education (15). The WHO has highlighted this initiative as a key goal, especially in low- and middle-income nations (16). In addition to the care given at each stage of dementia, the pandemic situation with the new coronavirus requires that everyone be protected. Some of the most vulnerable groups need special attention and care, namely, elderly people aged 60–65 years or older with CNCDs, such as dementia. Institutional care at all levels of public or private health strives to find a way to keep these individuals at home, in the safest possible way (9). The average age of this group is ~74 years, and their caregivers, many of them elderly, also need attention and care (17). The social isolation imposed by the pandemic makes it more difficult to attend outpatient follow-up consultations, and thus, a distance monitoring strategy may be useful. The objective of this study is to demonstrate the potential feasibility of a follow-up study with adjusted methodology for cost-effectiveness analysis, and we would like to clarify that this was the first virtual support model in our service.

We believe that this pilot study can stimulate other studies and will bring us more robust and consistent results for proposing partnerships for conducting collaborative studies of effectiveness in our country and in other countries as well. It is very important to comment here that despite our economic and social barriers, we understand that some low- and middle-income countries live with extreme economic and social inequalities that, at times, do not allow access to technologies that enable strategies such as the one presented in this work.

Even so, we believe that it is possible to create and or expand this kind of strategies with the participation of the health system, study centers, and neighborhood associations and improve existing actions in the primary care network with human and material resources that meet the needs of the population with these and other proposals that lead to health promotion and disease prevention actions, aiming at quality of life for patients with dementia and their caregivers and reduction of direct costs for the health system.

## Method

An observational pilot study was conducted involving non-pharmacological support interventions and online education to assist caregivers of dementia patients during the current pandemic. In fact, there are some important gaps in this section. However, in this pilot work, our main intention was to show that a virtual support for caregivers can make it possible to obtain positive results with education actions for the management with dementia at a distance. Our proposal was to identify whether there is a possibility to mitigate the direct social costs and the indirect costs of family caregivers. The intervention sought to help caregivers regarding their doubts and needs for information on health and disease management for patients followed at the HC-FMUSP Outpatient Clinic for Cognitive and Behavioral Neurology.

### Procedures

Potential participants were contacted by phone and 93 family caregivers were included. All participants were explained that due to social distancing measures, an educational and support activity would be provided, initially consisting of three weekly meetings each lasting 1 h. Two of the sessions would be conducted via virtual group meetings and one session would be to clear up doubts via WhatsApp messages. The messages received from caregivers via WhatsApp were monitored by the study coordinator, a specialist nurse in dementia. If an urgent/emergency issue was identified, a doctor in the group was contacted immediately to advise on the best approach; otherwise, the family member waited until a designated online meeting for the response. These meetings were structured as follows:

1. Mondays: Theme “Health Education.” Each system of the body was conceptualized separately, from an anatomic-functional perspective, along with the changes typical of senescence and the main diseases, with emphasis on advice for their prevention.
2. Fridays: Guidance on needs raised by the caregivers regarding the patient or themselves.
3. WhatsApp: Employed whenever caregiver deemed necessary where, in this case, the issues were first screened by the coordinator for urgency/emergency status and immediate response. If deemed non-urgent, caregivers waited until the next meeting on a specific day (Fridays).

After 3 months of online monitoring (Aug/Oct), an anonymous questionnaire was sent, also online, by agreement with the participating caregivers. The survey contained eight questions to collect information on the degree of satisfaction of caregivers in relation to the support offered. The study was part of the CAAD Project, approved by the Ethics and Research Committee of the Hospital das Clínicas of the Faculty of Medicine of the University of São Paulo under permit number 31041620.2.1001.0068 on November 5, 2020.

## Results

**Figure 2:**
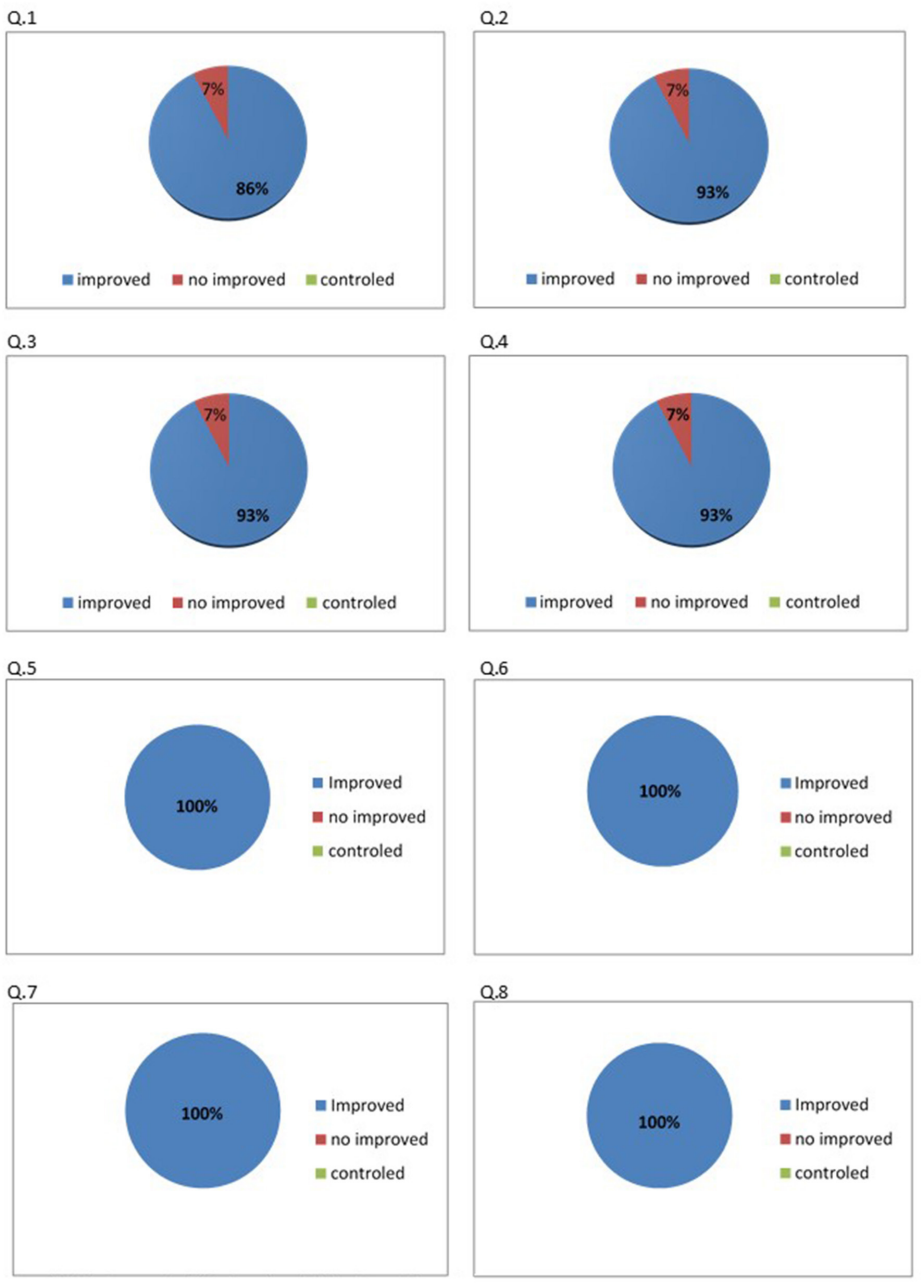
Preliminary results of virtual meetings GNCC-Support. Ferretti et al. (2020).

Of the total sample of caregivers (*N* = 93), only 45% (*n* = 42) answered the form sent containing the following questions: Q1. Do you think your daily life has improved after you started participating in the virtual meetings? Q2. Are you satisfied with the subjects of the virtual meetings? Q3. Do you now feel more secure with the support you are receiving? Q4. Are you practicing social isolation as recommended? Q5. Complies with health and hygiene measures aimed at preventing COVID-19 (hand washing, use of masks and alcohol gel, etc.)? Q6. Has the guidance given during the virtual meetings been useful? Q7. Did you feel welcome? Q8. Would you like this support to continue after the pandemic? The level of satisfaction among participants on questions 5 to 8 (100%) was high. On questions 2 to 4, the level of satisfaction ranged from 86 to 93%, while 7.1% of caregivers were dissatisfied with the program on each question. For one question (number 1), 86% of caregivers reported satisfaction with the program, 7.1% dissatisfaction, and 7.1% no change (Figure 2). Tables 1, 2 show the descriptive analysis of frequencies before and after the virtual program, and Table 3 shows the percentage improvement of difficulties to the caregivers after intervention model. Finally, Table 4 presents a Cochran model, a non-parametrical statistical analysis, used to show a global improvement of difficulties founded after the virtual support interventions.

**Table 1:** Frequencies of caregiver difficulties before the virtual support program.

	**Values**	
	^*^0	^#^1
$Q.2^**^B	42	0
$Q.3^**^B	42	0
$Q.1^**^B	42	0
$Q.4^**^B	42	0
$Q.5^**^B	42	0
$Q.6^**^B	42	0
$Q.7^**^B	42	0
$Q.8^**^B	42	0

*$Q, Question;*

* *0, difficulties of managements*;

** *B, Before*;

# *1 is considered as without difficulties*.

**Table 2:** Frequencies after the virtual support program.

	**Values**	
	0^**^	1^#^
^*^Q.2	6	36
^*^Q.3	6	36
^*^Q.1	12	30
^*^Q.4	6	36
^*^Q.5	0	42
^*^Q.6	0	42
^*^Q.7	0	42
^*^Q.8	0	42

* *Q, Question*;

** *0, difficulties of managements*;

# *1 is considered as without difficulties*.

**Table 3:** Percentage improvement data after the virtual support program.

***N*** **= 42**	**Improved (** ***n*** **)**	**%**	**Controlled (** ***n*** **)**	**%**
^&^Q.1	36	86	3	14
Q.2	39	93	3	7
Q.3	39	93	3	7
Q.4	39	93	3	7
Q.5	42	100	0	0
Q.6	42	100	0	0
Q.7	42	100	0	0
Q.8	42	100	0	0

& *Q.1–Q.8, Questions*.

**Table 4:** Cochran model for global improvement of difficulties after virtual support.

**Test statistics**			
***N***	**^a^Cochran's**	***df***	***p***
42	56,609	7	^#^0.000

# *p ≤ 0.001.*

a *Cochran's test*.

## Discussion

The study findings suggest that simple and very low-cost measures enabled the quality of non-pharmacological care to be maintained at this critical time via virtual support. Particularly during the current pandemic, everyone needs to be protected, while some more vulnerable groups need special attention and care, i.e., the elderly aged 60–65 years or older and those with CNCDs, such as dementia. A previous study published a few years ago explored the difficulties observed in low- and middle-income countries and showed that solutions can be found without the need for large investments when economic and social resources are limited (18), a finding corroborated by this pilot study. The training of professionals and of informal (family and friends) and formal (hired) caregivers on providing care has been discussed as an effective strategy that can prevent the onset of comorbidities and thus reduce healthcare costs for the State and the family (17, 18). In Brazil, for some years now, some isolated strategies have been employed that have proven the effectiveness of education programs in dementia, involving the use of printed support material at psychoeducational meetings, later delivered to caregivers or sent by email to those unable to attend face-to-face support meetings (19, 20). These studies have shown that patients and caregivers monitored in specialized services benefit from counseling measures and support groups focused on individualized needs, conferring numerous benefits in terms of both functional and behavioral aspects of patients with dementia, alleviating the burden of caregivers (21, 22).

These support materials, such as management and educational manuals that focus on information inherent to health promotion and disease prevention, are very useful in the primary healthcare network, helping both professionals and caregivers in the appropriate management of dementia, and their content can be delivered in person or remotely. Whether offered to lay caregivers or health professionals, the language of the content need only be adapted to the level of understanding of the participant. Good results can be obtained from non-pharmacological actions aimed at non-cognitive changes, activities of daily living, and caregiver stress (19, 21).

During this pandemic, several publications have shown results corroborating the effectiveness of virtual support, while others have noted their shortcomings (10, 23, 24). However, evidence on the socio-economic difficulties of low- and middle-income countries increasingly points to the use of strategies of this type. This is especially true when social distancing guidelines need to be observed more rigorously, when virtual interventions have proven to be excellent support measures, allowing clarifying of doubts, better organization of time spent on patient care, and prevention of problems related to the mental health of caregivers, who feel supported, albeit at a distance, by the professionals involved in the virtual support program (25). In a recent study, over 40% of caregivers reported clinically diagnosed depression, on average, 24 months after engaging in caregiving (17).

The burden on caregivers of dementia patients has always been high. In Brazil, the same study showed an average duration of 373 h (SD = 251.29 h) of informal patient care provision for basic activities of daily living, instrumental activities of daily living, supervision, and loss of productivity, translating to an average monthly monetary loss for the direct caregiver of US$118 (SD US$149.87), based on the February/2016 BRL–USD exchange rate (17).

In this pilot project, our positive expectations were confirmed, encouraging us to discuss and organize the methods to provide for the continuity of the program after the pandemic, as desired by the participating family members. It is now necessary to contextualize and confirm, by means of cost-effectiveness studies, that nursing interventions providing guidance on disease prevention in aging and on how, when, and why to adhere to certain individually prescribed procedures can improve the quality of life of patients and family members. This approach, especially amid the present health crisis, can cater to the needs of this high-risk group, which is more vulnerable to complications if infected by the new coronavirus, in societies still unprepared for this situation, such as Brazil. Currently, there are an estimated 1.5–1.7 million people living with dementia in Brazil and about 9 million in Latin America as a whole (25). Among all Latin American countries, Chile has proved the most compliant with the WHO proposals regarding the guidelines contained in the World Dementia Plan (16, 26). The Chilean model can contribute greatly to other Latin American countries still in the process of developing their national dementia plans. Despite the economic, social, and humanitarian difficulties, and in view of inequalities in LIMCs, as a social action, discussions should be encouraged among researchers working in diagnosis and treatment of dementias. This initiative could pave the way for a major forum in which each country can discuss its health policies and present suggestions that lead to a consensus for practical, objective, low-cost, and well-informed decision-making. This can provide the basis for a “pilot” intervention project involving all participating countries, whose results can be analyzed after 18 months, when its feasibility could then be discussed.

Recently, our Research Center at the Hospital of Clinics of the Faculty of Medicine of the University of São Paulo and other research centers technically supported, at the municipal level, the Alzheimer Law—number 17,547 approved on January 12, 2021. This Law primarily advocates a center of assistance, study, and research dedicated to early diagnosis, treatment, monitoring, and training of professionals and family members made by specialists. Our expectation is that this action may, in the future, be replicated throughout Brazil.

Until such initiatives can be implemented, education, and support strategies like that presented in this report can be useful in LMICs.

Economic and social difficulties that already existed in Brazil are now aggravated by the pandemic. At present, 14 million unemployed Brazilians are relying on assistance from government and NGOs, without any other source of income. The economic scenario is one of an annual increase in inflation of (IPCA) 4.31%, exceeding Brazil's Central Bank target for 2020 of 4%, a GDP that shows signs of recovery (7.7%), but insufficient to restore the economy in the country (27), and points to the need in public health for different ways of facilitating the delivery of information that can improve survival for patients with NCDs, and their caregivers, helping them maintain the quality of life of this group.

Finally, it may be useful to reflect that, if results (in terms of cost effectiveness) of a follow-on study prove promising for the groups involved, namely, a study group and control group (on waiting list), then perhaps:

- Many elderly caregivers can be spared the mobility difficulty of seeking non-pharmacological guidance in care units, even after the pandemic.
- Through health promotion and disease prevention efforts, health education can also reduce the pressure on emergency services with complaints that can be avoided by adequate guidance from the caregiver. Examples include cases of recurrent UTI admission for inadequate hygiene, dehydration as a result of low fluid intake, or constipation due to bad eating habits, low fluid intake, and inadequate diet.

Solutions that require little or no additional investment for these families likely to be implemented in the primary care network, as is the case with the online support system reported, need to be further explored and, given their accessibility, considered for adoption by primary care health systems of LMICs.

Managing modifiable risk factors is a measure that lends itself to online education programs. The preliminary findings observed in this study allow us to envisage that in the near future, some non-pharmacological support interventions online may be even more effective than face-to-face measures considering the context of dementia. In addition, the mobility difficulties encountered by family members to access referral centers, often to clarify doubts easily resolved remotely, may be overcome. Moreover, the burden generated not only by commuting, but also through formal work hours lost that, in many cases, are also online or informal, by the people who live with the patient, can expose these caregivers to several factors that negatively impact their socioeconomic and emotional status (18).

## Conclusion

The results of this very simple intervention suggest the utility of a program for caregivers of dementia patients in primary care. The intervention allowed a better understanding of the difficulties faced by caregivers in their daily care of dementia patients with daily management guidance given on a case-by-case basis. The program also promoted the implementation of an education strategy on the importance of understanding and recognizing anatomical and physiological changes in the aging process and their implications for the invisible line between senescence and senility. This empowers the caregiver to feel able to protect both the patient and themselves by preventing the emergence of common diseases in this age group.

Further studies are needed to explore this type of non-pharmacological support, which could prove to be an excellent and economical alternative for reducing direct and indirect costs related with dementia in LMICs. The new post-pandemic online support project provides for a longitudinal, cost-effective follow-up study that will prove the effectiveness of the non-pharmacological approach to nursing and that can control the emergence of common diseases in this age group, by reducing comorbidities and contributing to a healthy life throughout the life cycle. We envisage ongoing studies comparing these results and further demonstrating the potential for Latin America to reduce the direct and indirect burden on family members, promoting a more egalitarian and humanized social approach.

## Limitations

This is a low-cost strategy and can be an excellent possibility for professional and family interaction, increasing trust between them, which can lead to the construction of an economic and social model to support our society and other Latin American countries. The limitation of this pilot study is its design. At this point, we only intend to show through this pilot study a real and low-cost possibility for LMIC like Brazil. However, we performed a small descriptive analysis and also applied a non-parametric model to verify if this virtual assistance model was effective. We hope, in the near future, to expand this work to other Brazilian centers, so that we can compare the results obtained between us.

## Data Availability Statement

The original contributions presented in the study are included in the article/supplementary material, further inquiries can be directed to the corresponding author/s.

## Ethics Statement

The studies involving human participants were reviewed and approved by Ethics and Research Committee of Hospital das Clinicas, Faculty of Medicine, University of São Paulo. The patients/participants provided their written informed consent to participate in this study.

## Author Contributions

All authors listed have made a substantial, direct and intellectual contribution to the work, and approved it for publication.

## Conflict of Interest

The authors declare that the research was conducted in the absence of any commercial or financial relationships that could be construed as a potential conflict of interest.

## Publisher's Note

All claims expressed in this article are solely those of the authors and do not necessarily represent those of their affiliated organizations, or those of the publisher, the editors and the reviewers. Any product that may be evaluated in this article, or claim that may be made by its manufacturer, is not guaranteed or endorsed by the publisher.
